# A High-Density Admixture Scan in 1,670 African Americans with Hypertension

**DOI:** 10.1371/journal.pgen.0030196

**Published:** 2007-11-16

**Authors:** Rahul C Deo, Nick Patterson, Arti Tandon, Gavin J McDonald, Christopher A Haiman, Kristin Ardlie, Brian E Henderson, Sean O Henderson, David Reich

**Affiliations:** 1 Department of Genetics, Harvard Medical School, Boston, Massachusetts, United States of America; 2 Cardiology Division, Department of Medicine, Massachusetts General Hospital, Harvard Medical School, Boston, Massachusetts, United States of America; 3 Program in Medical and Population Genetics, Broad Institute of Harvard and Massachusetts Institute of Technology, Boston, Massachusetts, United States of America; 4 Department of Preventive Medicine, Keck School of Medicine, University of Southern California, Los Angeles, California, United States of America; 5 Genomics Collaborative, Cambridge, Massachusetts, United States of America; 6 Department of Emergency Medicine, Keck School of Medicine, University of Southern California, Los Angeles, California, United States of America; Baylor College of Medicine, United States of America

## Abstract

Hypertension (HTN) is a devastating disease with a higher incidence in African Americans than European Americans, inspiring searches for genetic variants that contribute to this difference. We report the results of a large-scale admixture scan for genes contributing HTN risk, in which we screened 1,670 African Americans with HTN and 387 control individuals for regions of the genome with elevated proportion of African or European ancestry. No loci were identified that were significantly associated with HTN. We also searched for evidence of an admixture signal at 40 candidate genes and eight previously reported linkage peaks, but none appears to contribute substantially to the differential HTN risk between African and European Americans. Finally, we observed nominal association at one of the loci detected in the admixture scan of Zhu et al. 2005 (*p* = 0.016 at 6q24.3 correcting for four hypotheses tested), although we caution that the significance is marginal and the estimated odds ratio of 1.19 per African allele is less than what would be expected from the original report; thus, further work is needed to follow up this locus.

## Author Summary

High blood pressure is more frequent and severe among African Americans than European Americans. To explore whether there are genetic underpinnings to this pattern, we screened the genomes of 1,670 African Americans, searching for loci at which people with hypertension (HTN) have more than the average proportion of African ancestry (eighty percent). We do not detect any region of clearly significant association. In a previous, smaller admixture scan for HTN genes, Zhu and colleagues (2005) reported two regions of association, which we would have expected to replicate if they were as strong as they initially appeared. While we detect marginal evidence of association at one, the signal is very weak, and much weaker than would have been expected from the previous report, so further work is necessary to understand this region. Our results are consistent with there being no common variants with a strong effect accounting for differences in HTN prevalence between African and European Americans. This increases the weight of evidence that non-genetic causes explain most of the difference in rates across populations.

## Introduction

Essential hypertension (HTN) is a widely prevalent condition with devastating clinical consequences including stroke, myocardial infarction, heart failure, and chronic kidney disease. Familial aggregation studies have established HTN as a complex trait, with both environmental and genetic determinants, and heritability for blood pressure has been estimated at about 30% [1]. Although HTN is prevalent worldwide (with particular abundance in developed countries), disease prevalence varies with ethnicity. In the United States, African Americans are disproportionately susceptible to HTN compared with other ethnic groups; they had an adjusted 1.6-fold higher prevalence of HTN than European Americans (and 2.5-fold higher than Mexican Americans) in the National Health and Nutritional Examination Survey (NHANES) in 2003–2004 [2]. The higher risk of HTN in African Americans has been hypothesized to be due (at least in part) to genetic risk variants that exist at a higher frequency in the ancestral African population than the ancestral European population [3]. Moreover, mean biochemical characteristics, such as plasma renin activity, urinary kallikrein and dopamine levels, differ between African Americans and European Americans with HTN, suggesting potentially different mechanisms of blood pressure elevation [4].

Admixture mapping is a technique that searches for genetic variants that differ strikingly in frequency between continental populations, and also contribute to disease. The idea of admixture mapping is to screen through the genome in a population of recently mixed ancestry, such as African Americans, identifying genome segments where in people with disease, there is a substantial deviation in the proportion of one of the parental ancestries from the genome-wide average. We specifically searched for regions with elevated African ancestry, based on the known higher rate of HTN in people of African ancestry.

Although the idea of admixture mapping is not new, it has only recently been implemented in practice with the introduction of panels of ancestry-informative markers [5,6] and statistical data analysis methods for detection of disease genes [7–10]. To date, four genome-wide admixture mapping scans have been published: one for HTN [11], one for multiple sclerosis [12], one for prostate cancer [13] and one for inflammatory biomarkers [14]. In addition to offering a novel method for identifying genetic determinants of HTN, admixture scans of HTN in African American may offer insights into the differences in salt-handling and blood pressure regulation between Africans/African Americans and European Americans; these differences are epidemiologically well-established and could be due to either genetic or non-genetic causes (see for example [15]).

## Results

For our analyses, we studied a data set of 1,165 individuals with HTN and 387 control individuals from the Multiethnic Cohort (MEC) [16] and a second data set of 505 individuals with HTN from the Genomics Collaborative (GCI) Study. The two studies differ in location of participants, method of data collection, and HTN phenotype definition (see Materials and Methods for study characteristics). Demographic characteristics for the two cohorts, as well as information on some important HTN covariates, are summarized in Table 1.

**Table 1:**
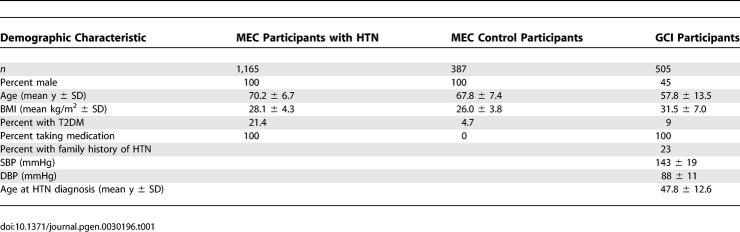
Demographic Characteristics for the Participants

The 1,670 individuals with HTN and 387 control individuals were genotyped using one of two panels of markers selected for high differences in frequency between West Africans and European Americans. A total of 911 individuals from the MEC were genotyped in the phase one panel, originally described in reference [12]. The remaining 641 MEC samples and 505 GCI samples were genotyped in a somewhat improved second-generation phase two panel, originally described in references [14,17].

### African Genetic Ancestry Is Not Significantly Higher in African Americans with HTN than in Control Individuals

The ANCESTRYMAP software [7] allows us to make precise estimates of individual African ancestry, which can be tested for correlation to HTN status. We find a trend towards increased African ancestry in hypertensive individuals (0.760) relative to control individuals (0.749) in the MEC; however, the result does not achieve statistical significance. The addition of the African/European ancestry term to a logistic regression model predicting HTN status that includes age, body mass index (BMI), and type II diabetes mellitus status (T2DM) also fails to significantly improve the fit of the model (unpublished data). Previous studies have also found a nonsignificant trend of HTN with increasing African ancestry [11,18],

### Admixture Mapping Detects No Significant or Suggestive Associations to HTN in Each Study Treated Separately

We carried out the genome-wide admixture scan by genotyping our 1,554 markers in the 1,670 individuals with HTN and 387 control participants. The resulting data were analyzed using two methods (Table 2): (a) an affected-only statistic, which calculates likelihood of association based on an estimate of the ancestry at a particular location relative to the overall average of the individual's genome (obviating the need for a separate control group), and (b) a case-control statistic, which measures the average ancestry deviation at a particular location in individuals with HTN and compares this with control participants [7]. While the affected-only statistic theoretically has more power to detect risk loci, the case-control statistic ensures that any deviation in African ancestry from the genome-wide average is present only in people with disease (for example, a locus unrelated to the disease could have been under selection some time in the recent history of the African American population, causing a rise in African ancestry relative to the average in the genome, irrespective of disease status).

**Table 2:**
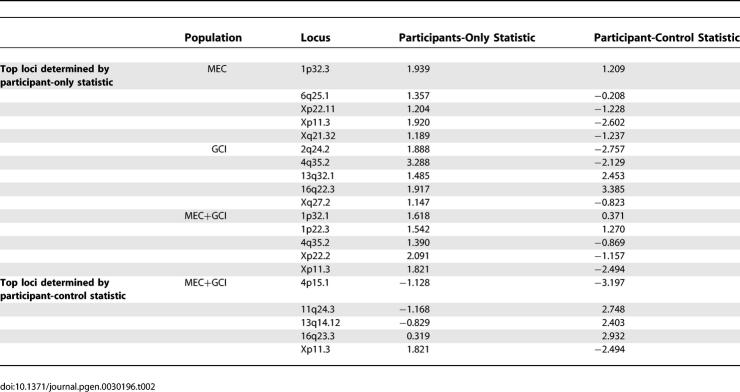
Top Loci Determined by HTN Participants-Only and Participant-Control Statistic for MEC, GCI, and Combined MEC+GCI Admixture Scans

**Figure 1:**
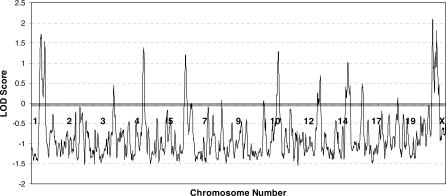
Genome-Wide Admixture Scans of a Total of 1,670 Participants with HTN and 387 Control Participants from GCI+MEC Do Not Reveal a Locus for Hypertension Risk The GCI+MEC combined scan yielded top LOD scores of 2.1 and 1.8 for peaks at Xp22 and Xp11 respectively, with a genome-wide score of −0.03.

We found no evidence for association to HTN. The genome-wide score, obtained by averaging the evidence of association at equally spaced points across the genome is −0.1 in the MEC and 0.6 for GCI, which does not meet our published thresholds of 1 for suggestiveness or 2 for significance [19]. The maximum local LOD score is 1.9 in the MEC and 3.3 in the GCI study (Table 2, Figure 1), again falling short of our published threshold of suggestiveness (LOD = 4) or significance (LOD = 5) [19].

### We Observed No Association with HTN after Combining Both Studies

Admixture mapping allows the merging of individuals with disease and control participants from multiple cohorts. This can increase the power to detect risk alleles with small effects, although merging data from cohorts collected in different ways can also weaken signals by combining heterogeneous phenotypes. We merged data from the MEC and GCI, yielding a total of 1,670 individuals with HTN and 387 control participants. The global genome-wide LOD score for the combined data was −0.03, indicating that the null hypothesis of no disease locus is slightly more favored than the hypothesis of a disease locus somewhere in the genome. The highest local LOD score applying the affected-only statistic was 2.09 on the X chromosome (Figure 1). The site on chromosome 4 with the highest LOD scores in the GCI scan showed a weakened LOD = 1.39 in the combined scan.

### We Found Little Evidence for Ancestry-Related Differences in HTN Risk at 40 Commonly Studied HTN Candidate Genes

One concern with whole genome scanning for disease variants is the risk of false negatives, with true disease variants buried underneath the noise of many loci, which necessitated a large correction for multiple hypothesis testing. One technique that has been proposed to deal with this problem is to pay special attention to candidate loci based on prior knowledge from linkage studies, association studies, and biological studies [20]. There have already been a handful of studies that mapped genes for extreme forms of HTN or hypotension [21], dozens of whole genome linkage scans for HTN, and thousands of candidate gene association studies [22,23]. In principle, taking these previous studies into account should help to prioritize signals in whole genome scans. To take advantage of this insight, we identified a small set of particularly plausible loci, to which we paid special attention even if the loci did not meet stringent thresholds of statistical significance correcting for scanning the whole genome.

We first carried out a focused analysis of 40 candidate genes previously identified in association studies (Table 3), and which we selected in three ways. First we searched PubMed (http://www.ncbi.nlm.nih.gov/sites/entrez?db=PubMed), the Genetic Association Database [24], and the HUGE Database (http://www.cdc.gov/genomics/hugenet) for genetic association studies involving HTN or blood pressure. We focused on genes with variants that demonstrated convincing association with HTN or blood pressure in two independent populations (see Materials and Methods for details of selection process). The genes we selected were primarily implicated in the renin-angiotensin-aldosterone axis, the adrenergic system, salt homeostasis, and T2DM. Second, we selected eight candidate loci based on their containing genes known to be causal in familial forms of HTN or hypotension; some of these overlap with those from the first category [21]. Third, we selected five candidate loci highlighted in a recent study by Young et al. [3], which focused on genetic variants implicated in salt handling and heat dissipation that show substantial worldwide frequency differences and marked differences between Sub-Saharan Africa and Northern Europe.

**Table 3:**
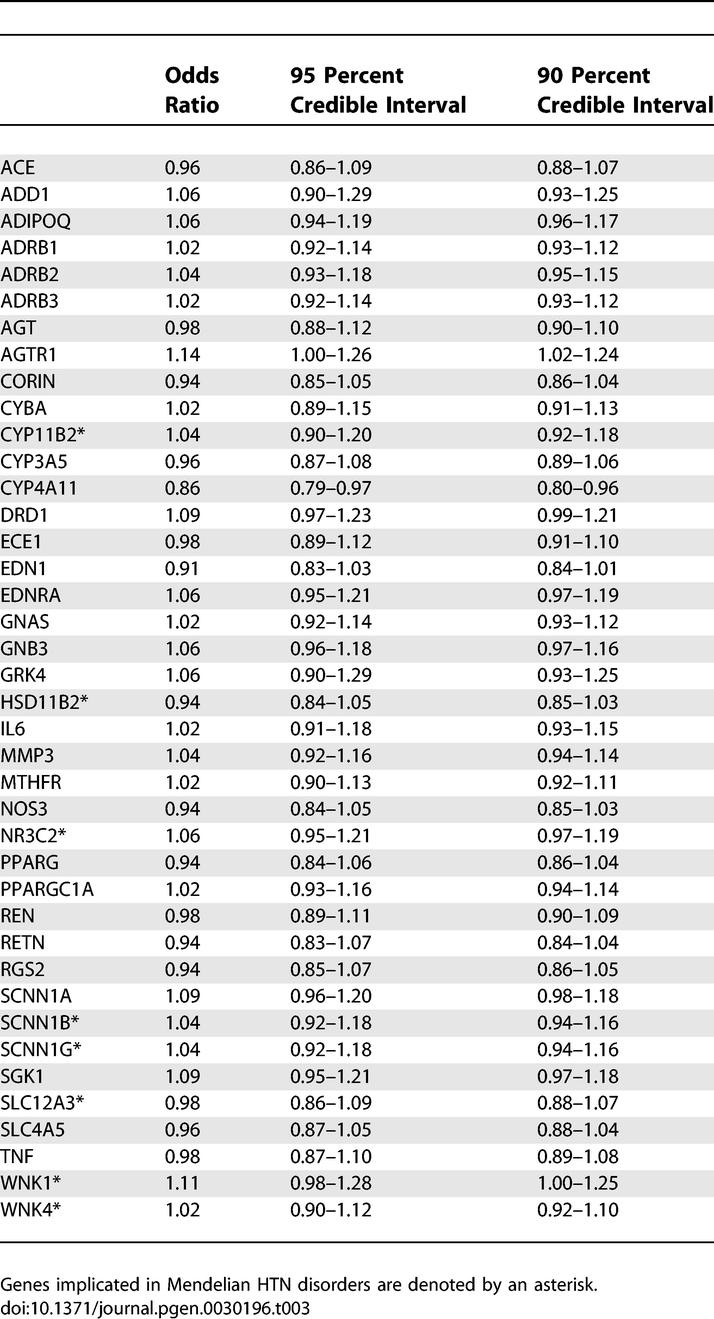
Odds Ratios (with 95% and 90% Credible Intervals) for HTN Risk Associated with the Inheritance of One African Ancestral Allele at 40 Plausible HTN Candidate Gene Loci

For each of the 40 genes, we calculated odds ratios (and credible intervals) for their admixture association to HTN risk in the combined GCI–MEC scan (Materials and Methods). Only three of these loci have a 90% credible interval that excludes no risk due to ancestry: *AGTR1, CYP4A11*, and *WNK1*. The *WNK1* and *AGTR1* genes both show increased HTN risk with inheritance of the African allele, which is the direction that might be expected given that African Americans have a higher rate of HTN (we estimate an increased risk for HTN of 1.11 and 1.14 for inheritance of one copy of the African allele at these loci, respectively). These signals of association are nonsignificant given the number of genes we tested. Moreover, because of the substantial extent of admixture linkage disequilibrium, even if these signals are real they may simply represent the effect of alleles of nearby genes rather than the proposed candidate genes. Admixture scanning of additional samples and other follow-up will be necessary to confirm these are genuine associations.

### No Overlap of Admixture Signals with Previously Reported Linkage Peaks

We also tested whether the admixture signals we found correlated with previously reported linkage peaks. Table 4 includes credible intervals for the top loci found in a recent meta-analyses of whole genome linkage scans for blood pressure traits [25–27]. In our admixture scan, none of these loci showed a significant association of ancestry with HTN risk.

**Table 4:**
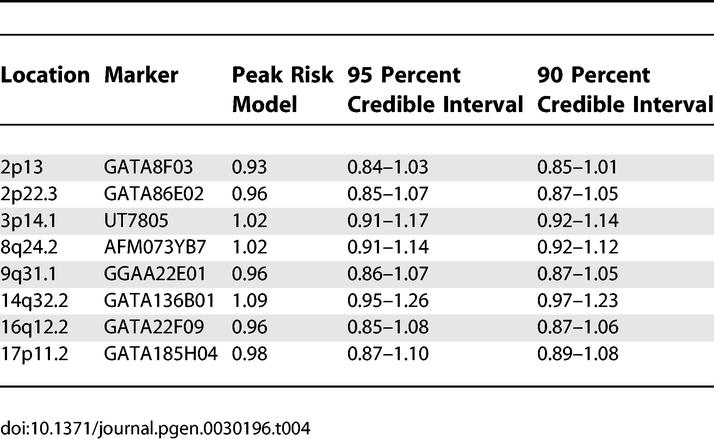
Odds Ratios (with 95% and 90% Credible Intervals) for HTN Risk Associated with the Inheritance of One African Ancestral Allele at Eight Plausible HTN Loci Identified from Meta-Analyses of HTN Linkage Scans

### One of the Four Strongest Loci in a Previously Published Admixture Scan for HTN Shows Evidence for an Increase in HTN Risk with African Ancestry

**Figure 2:**
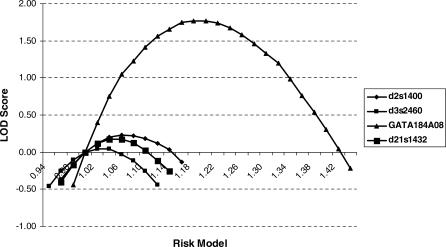
An Analysis of LOD Scores at Varying Risk Models Reveals Weak Evidence for Association of Previously Published Loci with HTN Using the distribution of LOD score shown below, we estimated a HTN odds ratio of 1.19 (95% credible interval 1.06–1.34) for inheritance of the African allele at the 6p24.1 (GATA184A08) locus, and odds ratios of 1.06 (0.94–1.21), 1.04 (0.95–1.17), and 1.02 (0.92–1.15) for the 2p25.1 (d2s1400), 21q21.1 (d21s1432), and 3q13.31 (d3s2460) loci, respectively.

Our scan can also be compared with the previous admixture mapping study for HTN, which identified two main candidate loci for association and two secondary loci [11]. We calculated odds ratios and credible intervals for all four of these peaks. Figure 2 demonstrates LOD scores for the 6q24.3, 2p25.1, 21q21, and 3q13.31 loci across a range of risk models. Overall, we find weak evidence for association with HTN for the 6q24.3 locus, with estimated odds ratios for HTN of 1.19 (95% credible interval 1.06–1.34, *p* = 0.004 by likelihood ratio test; *p* = 0.016 with Bonferroni correction for four hypotheses). For the 6q24.1 locus, there is an increased risk of HTN with inheritance of the African ancestral allele. This is the same direction that was also seen in the original report, although the quantitative effect is less than would have been necessary to generate the signal in reference [11].

### Ruling Out >95% of the Genome as Contributing Strong Risk Due to Ancestry

We constructed an exclusion map to rule out most loci in the genome as contributing substantially increased risk due to African or European ancestry. To obtain the exclusion map we estimated a confidence interval, for each position in the genome, for the factor by which the risk due to African (or European) ancestry differs from the risk due to European (or African) ancestry at that locus (Materials and Methods). For the 1,670 hypertensive individuals, 95.9% of the genome could be excluded as having risks > 1.3-fold due to African ancestry, and 98.7% could be excluded as having risks > 1.3-fold due to European ancestry at a significance of *p* < 0.05 (Table 5). The percentages of the genome excluded at various levels of risk are shown in Table 5. As our participants derive from two separate populations with different HTN definitions, we expect some loss of power from combining samples. We therefore also constructed an exclusion map with participants from the larger MEC cohort only. The percentage of the genome excluded using the MEC participants is shown in parentheses in Table 5, and reveals that, at a significance level of *p* < 0.05, 95.4% and 98.4% of the genome could be excluded as having risks > 1.3-fold due to African ancestry and European ancestry, respectively.

**Table 5:**
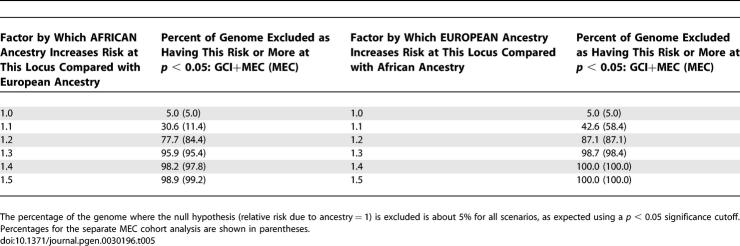
Proportion of Genome Excluded as Contributing to Differential Risk for HTN Comparing African and European Americans for Combined GCI+MEC and MEC-Alone Samples

### Inclusion of HTN Covariates Fails to Identify HTN Loci

Numerous covariates are known to affect blood pressure, including age, smoking, BMI, T2DM, and alcohol use. We considered the possible contribution of these covariates to the admixture association to HTN in two ways. Since T2DM is known to have an effect on blood pressure, we performed an admixture scan using only the 1,378 individuals with HTN but without T2DM from MEC and GCI. This revealed no significant or suggestive loci, with a genome-wide score of −0.1 and a peak LOD score of 1.8. We also attempted to address the presence of multiple covariates by constructing a logistic regression model for MEC HTN status including age, BMI, and T2DM, all of which are known HTN covariates, and all of which showed a significant association with HTN status in the MEC cohort. Using this model, we sought to identify surprising occurrences of HTN, which could not be accounted for by these three covariates. To do so, we determined the Pearson residual [28] for each individual, ranked participants in order of decreasing residual, and selected the top 25 percent of participants (highest residuals) for analysis. These should represent relatively lean, young, non-diabetic hypertensive individuals. Using the same regression model, we determined the Pearson residual for the GCI samples and for further analysis, and selected individuals who passed the cutoff defined in the MEC cohort. We then performed an admixture scan on the 482 individuals from MEC+GCI in the top 25 percent of HTN severity and compared them with 387 MEC control individuals. The genome-wide score is 0.0 (highest local LOD = 1.8), which does not reach statistical significance.

## Discussion

We have completed the largest and highest density HTN admixture scan to date and failed to identify any loci that are significantly, or even suggestively, associated with HTN after correcting for multiple hypothesis testing. Our large sample size also allows us to exclude > 95% of the genome as harboring risk loci of > 1.3 due to African or European ancestry. Although we cannot rule out weak ancestry effects, the negative results suggest that there may be no common variants with a strong effect accounting for differences in HTN prevalence between African and European Americans. These results thus increase the weight of evidence that non-genetic causes (diet and environment) contribute to the different epidemiology across populations.

An intriguing aspect of this study is the analysis of four loci from a previously reported admixture scan of HTN [11]. We observed nominal replication of the admixture association at one of these loci (*p* = 0.016 correcting for four hypotheses tested), and the direction of the association (increased HTN with increased African ancestry) is the same as previously reported. We caution that these results could represent statistical fluctuations, as numerous other loci in our scan scored more strongly. Another concern is that the estimate for the increased risk for HTN (1.19) arising from the inheritance of one African allele at this locus is sufficiently small that it would have been very surprising to observe genome-wide significant peaks in a scan with the sample size and map density studied by Zhu et al. [11]. Further follow-up studies will be necessary to properly test these loci for association. A possible way to reconcile the results from the two studies is that the samples in the Zhu et al. [11] study had a somewhat different phenotype than the ones we studied. Their definition of HTN, with multiple affected family members, may have been more genetically heritable, and thus their phenotype may have been more likely to yield association signals.

We also specifically evaluated risk at 40 biologically plausible HTN candidate gene loci as well as eight previously identified linkage peaks. For a complex disease, such as HTN, in which effects are expected to be weak, combining the wealth of prior genetic and biochemical data with whole genome scans may be essential for uncovering genes. Although our data highlight a small number of candidate loci, including the angiotensin Type I receptor and *CYP4A11*, independent studies will be needed for corroboration. We also examined five candidate genes that were highlighted by Young et al. [3], who proposed that HTN may arise from the interaction of salt-availability in humans populations with heat-adapted alleles that vary widely in frequency across populations. None of the five genes produced an admixture signal, suggesting that the underlying alleles do not explain a substantial amount of differential HTN risk across these populations.

A potential pitfall for our analysis is that we combined samples from two different studies, each with a different definition of HTN. The GCI study is based on physician-diagnosis, while the MEC, with its questionnaire-based data collection, obtains most information from patient self-report. To increase comparability across the two studies, we restricted our analysis of the MEC samples to individuals who reported using HTN-specific medications, guaranteeing that both the GCI and MEC samples were physician-treated individuals with HTN. We also performed additional analyses with non-diabetics only (since the percentage of participants with diabetes differed significantly in the two studies). We recognize that the difference in the phenotypes may reduce power for some analyses, as genetic determinants of HTN may not have the same effect in the two populations. In general, in our admixture mapping studies—not only for HTN, but also for prostate cancer [13] and multiple sclerosis [12]—we have taken an inclusive approach, analyzing as many individuals as possible that fit a loose definition of the phenotype, and following up marginal peaks by exploratory analysis across different subgroups. This was successful in identifying a locus for prostate cancer [13]; however, since it can also reduce power in some contexts, here we also present analyses of more homogeneous subgroups.

We conclude by noting that other factors may have contributed to our inability to identify HTN genetic variants by admixture mapping. It is possible that the phenotype definition we focused on was not sufficiently strong. Past successes at finding genetic risk factors for HTN have focused on families with extreme, familial forms of HTN, and here we aimed to find common variants affecting more commonly observed HTN in the community and the clinic [21]. For HTN, which is a classic complex trait, there are also a number of covariates that we did not consider, and that may have contributed to reduced power for detection of genetic determinants. In future admixture mapping and whole genome scans for HTN genes in African Americans, it will be particularly important to study samples that have been assessed not only for presence or absence of HTN, but also for differences in covariates that are known to differ across populations such as plasma renin activity, urinary kallikrein, and dopamine levels [4]. This may also offer insights into the differences in blood pressure and salt handling known to exist between African and European Americans [15].

## Materials and Methods

### Participants and control populations.

The samples in this study (*n* = 2,057) were all self-declared African Americans, and came from the California component of the MEC (*n* = 1,552) and from the GCI CARDIO study (*n* = 505). MEC is a National Cancer Institute funded prospective cohort of African Americans, Japanese Americans, Latinos, Native Hawaiians, and European Americans in California (mainly Los Angeles) and Hawaii. African Americans in this cohort were chosen by selecting census tracts in Los Angeles with a minimum percentage of individuals self-identified as African Americans in the 1990 census. Potential cohort members were identified through Department of Motor Vehicles drivers' license files and, for African-Americans, Health Care Financing Administration data files. Between 1993 and 1996, participants entered the cohort by completing a 26-page, self-administered (baseline) questionnaire that asked about diet, demographic factors, and history of prior medical conditions (e.g., HTN). In 2001, a short follow-up questionnaire was sent to update information on specific dietary habits as well as to obtain information about new diagnoses of medical conditions since recruitment. In the MEC, hypertensive individuals were defined as those who indicated on the baseline questionnaire that they had a history of HTN, and had taken or were taking antihypertensive medications, and who reported themselves as hypertensive on the follow-up questionnaire. Normotensive control participants were defined as those who answered no to all of the above questions [16]. The GCI collection consisted of individuals referred by primary care physicians or specialists. For patients to be regarded as hypertensive, they required at least two documented blood pressures > 140/90 prior to the initiation of anti-hypertensive medication. If blood pressures prior to initiation of medication were not available, then the patients required two documented blood pressures > 140/90 while under treatment.

### Selection of SNPs for the admixture panel.

The phase one panel (911 samples in our study) consisted of 1,824 SNPs, mostly overlapping the set described in reference [12]. The core of this panel consisted of 1,536 SNPs chosen from the map published in [5] and genotyped using the Illumina GoldenGate platform [29]; we then supplemented this panel with an additional 288 SNPs genotyped on the Sequenom MassArray and iPLEX platforms [30] to fill in gaps in the map and increase density at regions of high interest. The phase two panel (1,146 samples in our study) consisted of 1,566 SNPs, largely overlapping the panel described in reference [14]. This panel was constructed by supplementing the database of ancestry informative markers reported on in Smith et al. [5], with > 1,500 new markers selected from Hinds et al. [31] as likely to be highly informative about ancestry and to fill in gaps in the phase one panel. The final phase two panel of SNPs was chosen as the most informative 1,536 SNPs from the extended database (genotyped on the Illumina GoldenGate platform), supplemented by 30 SNPs genotyped using the Sequenom MassArray and iPLEX platforms [30] to increase density in regions of high interest.

### Frequency estimates from the ancestral populations.

To obtain frequency estimates for each of the SNPs in Africans and Europeans, we used previously published data [5,13]. West African ancestral frequencies were estimated using samples from Ghana (*n* = 33), Cameroon (*n* = 20), and Nigeria (*n* = 122). European frequencies were obtained using samples from Baltimore (*n* = 38), Chicago (*n* = 39), Italy (*n* = 42), Poland (*n* = 47) and Utah (*n* = 93). These samples provided a Bayesian prior distribution for the allele frequencies in the parental populations as described in reference [7].

### Whole-genome amplification of DNA.

All the MEC samples were subjected to whole-genome amplification (Molecular Staging) to produce sufficient DNA sufficient for genotyping [32].

### Elimination of poorly performing samples.

DNA samples were excluded if they showed (a) less than 85% genotyping success rate, or (b) a striking excess or deficiency of heterozygote genotypes compared with that expected from the individual's estimated proportion of European ancestry. The high heterozygosity filter is aimed at removing individuals who have one European parent, and who are not correctly modeled by the ANCESTRYMAP software so that analysis of these samples might weaken statistical power [7]. The low heterozygosity filter is aimed at removing individuals with low quality DNA and high genotyping error rate.

### SNP genotyping and quality control.

We used a series of criteria for eliminating SNPs from the analyses. We started with two panels of SNPs, designated phase one consisting of 1,824 SNPs and phase two consisting of 1,566 SNPs. A total of 760 SNPs were common to both panels, leaving 2,630 unique SNPs. Individuals were genotyped in either the phase one or phase two panel, but not both. We eliminated SNPs from the analysis by including only those with > 85% genotyping success rate in African American HTN participants, and that had reliable genotype clustering patterns as judged by an experienced research technician (GJM). This left 2,007 SNPs. We next imposed a requirement for Hardy-Weinberg equilibrium (*p* > 0.01) in both the ancestral West African or European American populations. Finally, we required that the frequency in African American control participants was appropriately intermediate between ancestral West Africans and European Americans [7]. This left 1,966 SNPs. We also eliminated SNPs that demonstrated linkage disequilibrium in the parental populations, as these are liable to produce false positive signals of association [19]. After these filters, we were left with 1,554 markers useable for analysis.

### Risk model used in the Markov Chain Monte Carlo data analysis.

The ANCESTRYMAP software described in [7] was used for all analyses. The program combines information from multiple, densely spaced markers that are each partially informative about African versus European ancestry, to produce robust, multipoint estimates. The LOD score for association is defined as the log ratio of the likelihood of the data under a disease model, divided by the likelihood of the data under no disease model. We evaluated LOD scores at equally spaced points across the genome. At each point, we used a multiplicative model of risk, with risk of disease integrated over the inheritance of 0, 1, and 2 copies of an African ancestral allele. By the convention used in the manuscript, a risk > 1.0 for inheritance of one African ancestry allele at a given locus describes a model where African ancestry increases disease risk relative to European ancestry. The ANCESTRYMAP software uses Bayesian statistics and thus requires specification of a prior distribution on risk models before carrying out the analysis. We ran a range of risk models for the MEC, GCI, and MEC+GCI samples, and averaged the LOD score at equally spaced points in the genome (one point every centimorgan). The prior distribution we used was a range of ten risk models from 1.6-fold increased risk due to inheritance of one African ancestral allele to 1.6-fold increased risk due to inheritance of one European allele.

### Selection of candidate HTN genes.

We used three overlapping sources to identify candidate genes: PubMed (http://www.ncbi.nlm.nih.gov/sites/entrez?db=PubMed), the Genetic Association Database [24], and the Human Genetic Epidemiology (HUGE) Database (http://www.cdc.gov/genomics/hugenet). We searched PubMed for all references mentioning association of genetic variants with HTN or blood pressure (details of search terms are available upon request). We performed similar searches using the Genetic Association Database and the HUGE database. We identified over two hundred genes for which variants had been tested for association with HTN. From these, we selected genes that had positive associations for at least one variant with blood pressure or HTN in two or more populations. We restricted ourselves to association studies with at least 100 participants with HTN and 100 control participants or 200 total individuals for quantitative trait evaluation. For each candidate gene, we identified the genetic position using Build 35 of the public genome reference sequence (http://genome.ucsc.edu) and selected the closest marker to estimate the ancestry-associated HTN risk at that locus.

### Exclusion map.

To obtain credible intervals for increased risk due to African ancestry at candidate loci in the genome, we carried out repeated runs of ANCESTRYMAP, each testing for a different disease model. The analysis was repeated for 65 disease models, consecutively running the analysis software for disease risk models of 0.40, 0.42, 0.44, 0.46 …, 1.66, 1.68 and 1.70-fold increased risk due to one African allele, and searching for the maximum likelihood risk model. The 90% and 95% credible intervals for increased risk due to African ancestry were obtained by a likelihood ratio test: as the range of models for which the log base ten of the likelihood of the disease model was within 0.588 and 0.883 of the maximum (we used interpolation to extract the disease risk model, accurate to two decimal places, that met this criterion).

### HTN logistic regression model.

A logistic regression model was developed to predict HTN status using the 1,165 MEC HTN participants and 387 MEC HTN control participants, with age, BMI, and T2DM status as independent variables. Full covariate information was available for 1,132 MEC participants with HTN and all 387 MEC control participants. Using coefficients determined from the model, the Pearson residual [28] was calculated for each individual. Individuals were ranked by residual, and the top 25% of individuals were selected for the analysis. Using the same coefficients, a regression residual was also calculated for the 505 GCI participants, and the 199 individuals with residuals above the same cutoff were selected for a combined admixture scan with the 283 MEC participants and 387 MEC control participants. All analyses were performed using Stata 8.0 (StataCorp).

### Online resources.

See http://genepath.med.harvard.edu/~reich for our ANCESTRYMAP software.

## Supporting Information

### Accession Numbers

Accession numbers for genes mentioned in this paper from the Entrez GeneID database (http://www.ncbi.nlm.nih.gov/sites/entrez?db=gene) are ACE (1636); ADD1 (118); ADIPOQ (9370); ADRB1 (153); ADRB2 (154); ADRB3 (155); AGT (183); AGTR1 (185); CORIN (10699); CYBA (1535); CYP11B2 (1585); CYP3A5 (1577); CYP4A11 (1579); DRD1 (1812); ECE1 (1889); EDN1 (1906); EDNRA (1909); GNAS (2778); GNB3 (2784); GRK4 (2868); HSD11B2 (3291); IL6 (3569); MMP3 (4314); MTHFR (4524); NOS3 (4846); NR3C2 (4306); PPARG (5468); PPARGC1A (10891); REN (5972); RETN (56729); RGS2 (5997); SCNN1A (6337); SCNN1B (6338); SCNN1G (6340); SGK1 (6446); SLC12A3 (6559); SLC4A5 (57835);TNF (7124); WNK1 (65125); and WNK4 (65266).

## Abbreviations

BMI: body mass index
HTN: hypertension
MEC: multiethnic cohort
GCI: Genomics Collaborative
T2DM: type II diabetes mellitus
